# Lock and dock: Two-step transvenous retrieval of a fractured femoral sheath with a vascular snare via the right internal jugular vein

**DOI:** 10.1016/j.jccase.2023.06.007

**Published:** 2023-07-04

**Authors:** Yuhei Kasai, Jungo Kasai, Takuya Haraguchi, Takayuki Kitai, Junji Morita, Takuya Okada, Masanaga Tsujimoto, Tsutomu Fujita

**Affiliations:** a Department of Cardiology, Asia Medical Group, Sapporo Heart Center, Sapporo Cardiovascular Clinic, Sapporo, Japan; b Paul G. Allen School of Computer Science & Engineering, University of Washington, Seattle, WA, USA; c Department of Clinical Engineering, Asia Medical Group, Sapporo Heart Center, Sapporo Cardiovascular Clinic, Sapporo, Japan

**Keywords:** Fractured sheath, Vascular snare, Internal jugular vein approach, Large-diameter sheath, Catheter ablation

## Abstract

An 86-year-old male with progressive palpitations and dyspnea was referred to our hospital for heart failure treatment. Catheter ablation was performed for atrial flutter as we suspected tachycardia-induced cardiomyopathy as the cause of the patient's heart failure. Due to difficulty securing a peripheral venous route, a 6-Fr sheath was inserted via the right common femoral vein prior to administering general anesthesia. While attempting to insert a mapping catheter, the 6-Fr sheath became lodged and subsequently fractured during removal. Percutaneous transvenous retrieval using an 8-Fr sheath was unsuccessful, and a switch to a right internal jugular vein approach with a 16-Fr sheath was necessary for successful retrieval. The following two-step retrieval (“lock and dock”) was then performed: 1) lock: a vascular snare was used to catch the remaining wire crossing into the fractured sheath lumen to prevent the risk of sheath migration to the right ventricle or the pulmonary artery, and 2) dock: the same snare was subsequently used to catch the fractured sheath. The planned catheter ablation was then successfully performed, without any complications.

**Learning objective:**

Our case presents, “lock and dock,” a novel approach for percutaneous transvenous retrieval that involves two steps: a vascular snare is used to catch the wire and subsequently the fractured sheath. This use of a vascular snare and a large-diameter sheath through the right internal jugular vein effectively reduces the possibility of fractured sheath migration.

## Introduction

As the number of percutaneous procedures increases, there will also be an increase in the number of cases of fractured sheath retrieval. Percutaneous endovascular retrieval is preferred to invasive, open surgery. Several retrieval techniques using a vascular snare have been reported [1]. When performing percutaneous retrieval, the risk of fractured sheath migration to the right ventricle (RV) and the pulmonary artery (PA) is a significant concern. This is the first report of successful percutaneous retrieval using a right internal jugular vein (IJV) approach with the following two steps (“lock and dock”): a vascular snare was first used to catch the remaining wire crossing into the fractured sheath lumen to prevent the risk of sheath migration (lock), and the same snare was subsequently used to catch the fractured sheath (dock).

## Case report

**Fig. 1:**
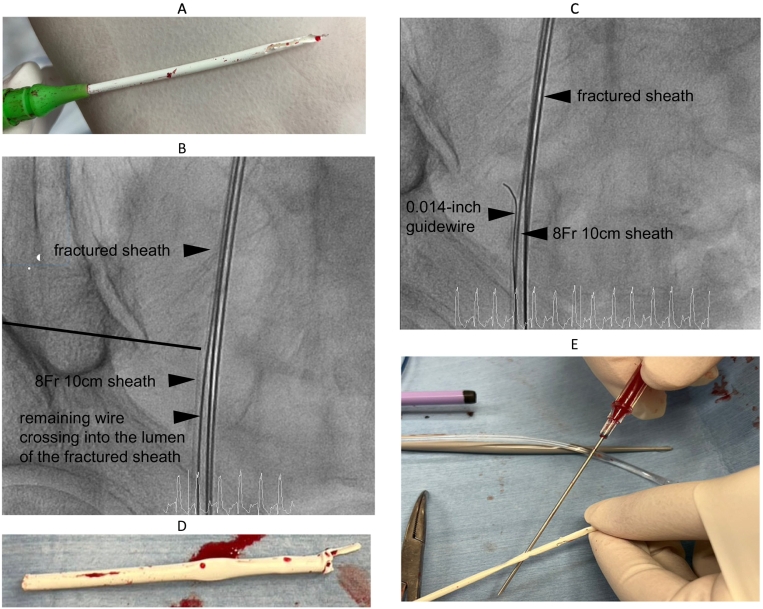
(A) Image of the proximal portion of the fractured sheath. (B) The 8-Fr sheath advanced to the distal portion of the fractured sheath. (C) Unsuccessful crossing of the 0.014-in. guidewire into the lumen of the fractured sheath. (D) Image of the extracted sheath fragment (the distal portion of the fractured sheath). (E) Additional experiment that shows our puncture needle can pass through the 6-Fr sheath.

An 86-year-old male who experienced progressive palpitations was referred to our hospital for heart failure. Because tachycardia-induced cardiomyopathy due to atrial flutter (AFL) was suspected as the cause of heart failure, we decided to perform catheter ablation for AFL. With the patient's consent, catheter ablation was performed under general anesthesia. At the beginning of the procedure, venous access was planned in the right common femoral vein (CFV) using an 8.5-Fr deflectable sheath (Vizigo sheath; Biosense Webster Inc., Irvine, CA, USA), an 8.5-Fr sheath (Swartz sheath; St. Jude Inc., St. Paul, MN, USA), and a 10-cm 6-Fr sheath (Terumo, Tokyo, Japan). The intravenous line leaked and there was insufficient anesthesia, so a 6-Fr sheath was inserted after single puncture. The remaining two punctures were then performed. Afterwards, while attempting to insert a duodecapolar catheter (Biosense Webster Inc.) into the 6-Fr sheath, it became lodged. The sheath was likely kinked, so we attempted to insert an inner tube and wire, but although the wire reached the IVC, the inner tube did not advance. While attempting to completely remove the 6-Fr sheath, the terminal 6-cm tip of the sheath detached within the right CFV during venous withdrawal (Fig. 1A).

We initiated percutaneous transvenous retrieval of the fractured 6-Fr sheath. We confirmed that the wire was passed through the lumen of the fractured sheath using multidirectional fluoroscopic guidance. First, a 10-cm 8-Fr sheath (Terumo) was carried to the front of the fractured sheath over the remaining wire crossing into the fractured sheath lumen. The 8-Fr sheath was advanced to the distal portion of the fractured sheath (Fig. 1B). Subsequently, we attempted to cross a 0.014-in. guidewire (Jupiter FC; Boston Scientific, Marlborough, MA, USA) that had been inserted from the 8-Fr sheath into the lumen of the fractured sheath. However, it became twisted, preventing successful crossing into the lumen of the fractured sheath (Fig. 1C). Considering the risk of migration to the RV and PA, we attempted fractured sheath retrieval through the right IJV by grasping the remaining wire crossing into the fractured sheath lumen. A large-diameter (16-Fr) sheath (Check-Flo Performer; Cook Medical, Bloomington, IN, USA) was inserted into the right IJV to facilitate fractured sheath retrieval. We also considered the left femoral approach, but we suspected that it would be easier to grasp the tip of the guidewire, which passes through the fractured sheath, from the inferior vena cava (IVC) on the central side of the iliac vein bifurcation. There is also a possibility that there are holes in the sheath other than the torn part, and we wanted to minimize the burden on the fractured sheath to avoid further fracture.

**Fig. 2:**
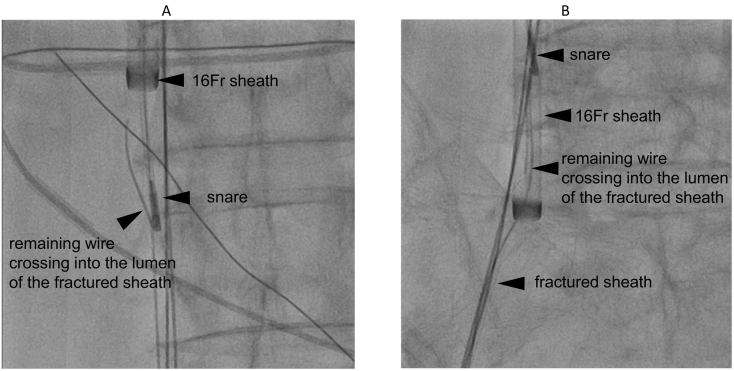
The first step of percutaneous transvenous retrieval (lock). (A) The remaining wire crossing into the fractured sheath lumen grasped by the snare. (B) The remaining wire crossing into the fractured sheath lumen was retrieved into the 16-Fr sheath.

Our first step (lock) reduces the risk of fractured sheath migration into the RV and PA. Specifically, the wire crossing into the lumen of the fractured sheath was grasped using the ONE SNARE™ (Merit Medical, South Jordan, UT, USA) inserted into the 16-Fr sheath (Fig. 2A). Afterwards, the wire, which crossed into the fractured sheath lumen, was retrieved into the 16-Fr sheath as the 16-Fr sheath was carried to the right common iliac vein (Fig. 2B).

**Fig. 3:**
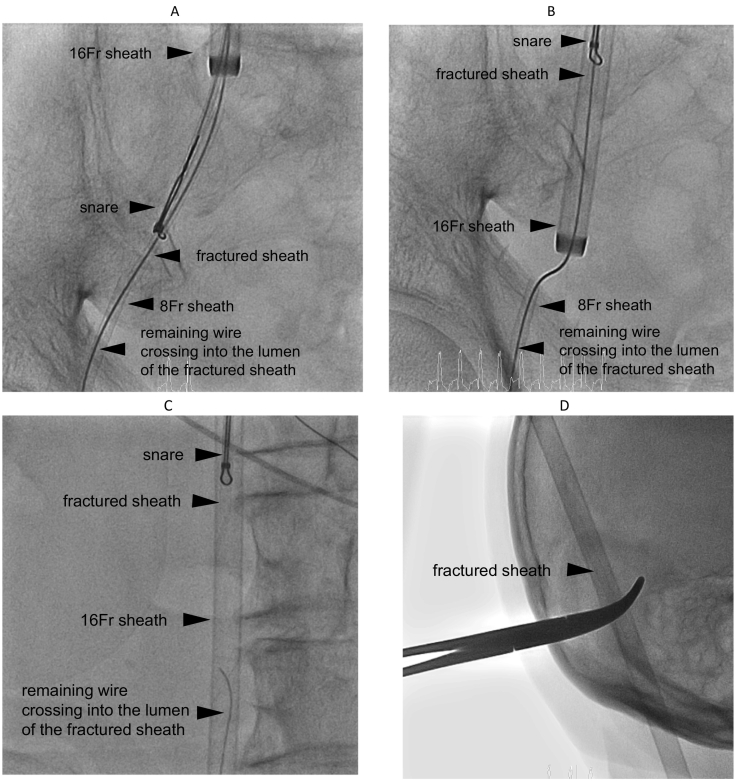
The second step of percutaneous transvenous retrieval (dock). (A) The fractured sheath grasped by the snare. (B) Success of fractured sheath retrieval with the 16-Fr sheath. (C) Retrieval of only the fractured sheath, not the remaining wire. (D) Fractured sheath removal along with the 16-Fr sheath.

As the second step (dock), the fractured sheath was grasped by the same snare, which was then pulled (Fig. 3A, B, C; Video 1, Video 2). Once the fractured sheath had been pulled out to the external part of the 16-Fr sheath, it was clamped with forceps and removed, along with the 16-Fr sheath (Fig. 3D). The extracted sheath fragment was examined to confirm that the entire fractured sheath had been removed (Fig. 1D). The planned catheter ablation was then performed, resulting in a return to sinus rhythm.

## Discussion

Fractured catheters that cannot be removed can cause serious complications, such as embolization and vessel perforation. The first-choice method for removing fractured catheters is percutaneous retrieval [2,3]. Most cases of broken catheter retrieval that involve the venous system (as in the present case) migrate to the RV and PA during percutaneous retrieval. Fractured sheath embolization may lead to cardiac rupture, various arrhythmias, and pulmonary hypertension [4]. Therefore, it is crucial to prevent the fractured sheath from migrating to the RV and PA.

It is suspected that puncture needles for other sheaths may have passed through the 6-Fr sheath, causing it to become weakened or compromised. We performed an additional experiment and found that our puncture needle can indeed pass through the 6-Fr sheath (Fig. 1E). After inserting the 6-Fr sheath and administering propofol and fentanyl intravenously through the 6Fr sheath, we punctured the proximal part, relative to the 6-Fr sheath. We had difficulty with the puncture and tried several times, and we might have accidentally pierced and damaged the 6-Fr sheath with the needle during the difficult puncture.

For sheath retrieval from the right CFV, it is necessary to insert a sheath that is thicker than the broken 6-Fr sheath by placing the wire crossing into the fractured sheath lumen. However, there is a risk that the fractured sheath will slip off the wire if the position of the wire moves, making it difficult to insert a thicker sheath. Moreover, the remaining wire crossing into the lumen of the fractured sheath only protruded approximately 12 cm out of the body. The thickest available one was a 10 cm 8-Fr sheath. Our plan was to advance a balloon on the 0.014-in. wire through the fractured sheath into the IVC, inflate it, and then retract the fractured sheath along with the 8-Fr sheath and the balloon. We were, however, unable to cross the 0.014-in. guidewire into the lumen of the fractured sheath because of the structure of the broken sheath (stretched part in Fig. 1D). We then switched to the right IJV approach with the snare, and a 16-Fr sheath was inserted through the right IJV. We chose the right IJV instead of the left CFV based on the coaxiality. Both the snare and the fractured sheath were also 6 Fr; thus, theoretically a large-diameter sheath of ≥12-Fr was required for retrieval. We chose a sheath with a large diameter (16-Fr) to ensure easy retrieval. In a previous study, a 23-Fr sheath was inserted through the right IJV for leadless pacemaker implantation without any vascular complications [5], suggesting that the 16-Fr sheath is a reasonable choice.

Nevertheless, internal jugular catheterization generally carries risks of mechanical complications such as pneumothorax, hemothorax, and arteriovenous fistula associated with arterial puncture [6]. Additionally, a previous report has shown that mechanical complications can also occur when using dilators for insertion of large-diameter sheaths [7]. The use of ultrasound guidance has been shown to reduce the risk of these mechanical complications [8]. Considering these risks, we performed the right IJV puncture under ultrasound guidance and carefully inserted the dilator and sheath while monitoring the fluoroscopic image.

Generally, the success rate of retrieval depends on whether there is a wire in the fractured sheath lumen. Advanced techniques, such as balloon-supported retrieval, are necessary when the broken sheath departs from the wire [9]. These advanced techniques are associated with a crucial risk of sheath migration and vein rupture caused by damage to the venous wall. Retrieval from the original puncture site (the right CFV) is associated with such a risk because the fractured sheath can slip off during retrieval. The right IJV approach used in this study caught the wire by the snare inserted through the right IJV in the first step (lock), thereby greatly reducing the risk of sheath migration. We first inserted an 8-Fr sheath from the right femoral and attempted to cross the 0.014-in. guidewire into the lumen of the fractured sheath. This procedure would avoid additional puncture but increase the risk of the fractured sheath slipping off the guidewire. We therefore recommend retrieval through the right IJV as the first choice in practice.

## Conclusion

We presented “lock and dock,” a new, two-step method for percutaneous retrieval of an intravenous fractured sheath. The technique involves using a vascular snare to capture the wire and then the fractured sheath. The first step of catching the wire with the snare (lock) greatly reduces the risk of sheath migration.

The following are the supplementary data related to this article.

Video 1 Video 1

Video 2 Video 2

## Declaration of competing interest

None.
